# Interactions between Amyloid-β and Hemoglobin: Implications for Amyloid Plaque Formation in Alzheimer's Disease

**DOI:** 10.1371/journal.pone.0033120

**Published:** 2012-03-06

**Authors:** Jia-Ying Chuang, Chu-Wan Lee, Yao-Hsiang Shih, Tingting Yang, Lung Yu, Yu-Min Kuo

**Affiliations:** 1 Institute of Basic Medical Sciences, National Cheng Kung University, Tainan, Taiwan; 2 Division of Neuroscience and Neuropathology, The School of Chinese Medicine for Post-Baccalaureate, I-Shou University, Kaohsiung, Taiwan; 3 Institute of Behavioral Medicine, National Cheng Kung University, Tainan, Taiwan; 4 Department of Cell Biology and Anatomy, National Cheng Kung University, Tainan, Taiwan; Massachusetts General Hospital/Harvard Medical School, United States of America

## Abstract

Accumulation of amyloid-β (Aβ) peptides in the brain is one of the central pathogenic events in Alzheimer's disease (AD). However, why and how Aβ aggregates within the brain of AD patients remains elusive. Previously, we demonstrated hemoglobin (Hb) binds to Aβ and co-localizes with the plaque and vascular amyloid deposits in post-mortem AD brains. In this study, we further characterize the interactions between Hb and Aβ in vitro and in vivo and report the following observations: 1) the binding of Hb to Aβ required iron-containing heme; 2) other heme-containing proteins, such as myoglobin and cytochrome C, also bound to Aβ; 3) hemin-induced cytotoxicity was reduced in neuroblastoma cells by low levels of Aβ; 4) Hb was detected in neurons and glial cells of post-mortem AD brains and was up-regulated in aging and APP/PS1 transgenic mice; 5) microinjection of human Hb into the dorsal hippocampi of the APP/PS1 transgenic mice induced the formation of an envelope-like structure composed of Aβ surrounding the Hb droplets. Our results reveal an enhanced endogenous expression of Hb in aging brain cells, probably serving as a compensatory mechanism against hypoxia. In addition, Aβ binds to Hb and other hemoproteins via the iron-containing heme moiety, thereby reducing Hb/heme/iron-induced cytotoxicity. As some of the brain Hb could be derived from the peripheral circulation due to a compromised blood-brain barrier frequently observed in aged and AD brains, our work also suggests the genesis of some plaques may be a consequence of sustained amyloid accretion at sites of vascular injury.

## Introduction

Alzheimer's disease (AD) is characterized pathologically by the presence of a large number of intraneuronal neurofibrillary tangles, extracellular amyloid plaques and cerebral amyloid angiopathy together with the loss of synapses. The main component of the amyloid plaques and cerebral amyloid angiopathy is the amyloid-β (Aβ) peptide, derived from a larger amyloid precursor protein (APP), by the proteolytic actions of β-secretase and γ-secretase [1], [2]. Neuronal injury (e.g. dystrophic neurites and synaptic loss) and microglia activation in the vicinity of amyloid plaques led to the proposition of the amyloid cascade hypothesis– profuse accumulation of soluble and insoluble forms of Aβ as the major cause of dementia [3], [4]. This tenet was reinforced by the engineering of APP transgenic mice and the discovery of early-onset familial forms of AD [3], [4]. The neurotoxicity of certain aggregated forms of Aβ (e.g. oligomeric and protofibrillar) further strengthens the view of Aβ as a key AD culprit [5]–[8].

Although extensive investigations have been implemented, the mechanism for the abnormal accumulation and deposition of Aβ peptides in the majority of sporadic late-onset AD brains remains unclear. Soluble forms of Aβ exist in normal human brains and CSF [9], [10] and except for a few familial AD cases, the expression of APP and production of Aβ are not increased in most sporadic AD patients [11]–[13]. In contrast, hypoxic/ischemic conditions and traumatic brain injuries up-regulate APP mRNA and protein [14]–[17] suggesting potential physiological roles for APP and/or Aβ. Furthermore, numerous molecules are known to interact with Aβ and alter the folding, stability and transport/clearance of these peptides [18]–[22].

We and others have shown that hemoglobin (Hb) binds to Aβ and co-localizes in amyloid plaques and cerebral amyloid angiopathy in AD brains [23], [24]. However, the mechanisms for the interaction between Aβ and Hb and the potential roles of such interaction remain unclear. In this study, we initially characterized the binding between Aβ and Hb by incubating Aβ with different components of Hb: 1) apohemoglobin (apoHb) or heme-free Hb, 2) heme and 3) protoporphyrin IX (PpIX) ring or iron-free heme. In addition, the independent neurotoxicity of heme and Aβ or a mixture of both molecules was examined in vitro. The expression patterns of Hb in the brains of APP/PS1 transgenic (tg) mice and post-mortem AD patients were also assessed. Finally, the in vivo interaction between Hb and Aβ was studied by injection of Hb into the hippocampi of APP/PS1 tg mice.

## Results

### Interaction between Hb and Aβ

**Figure 1:**
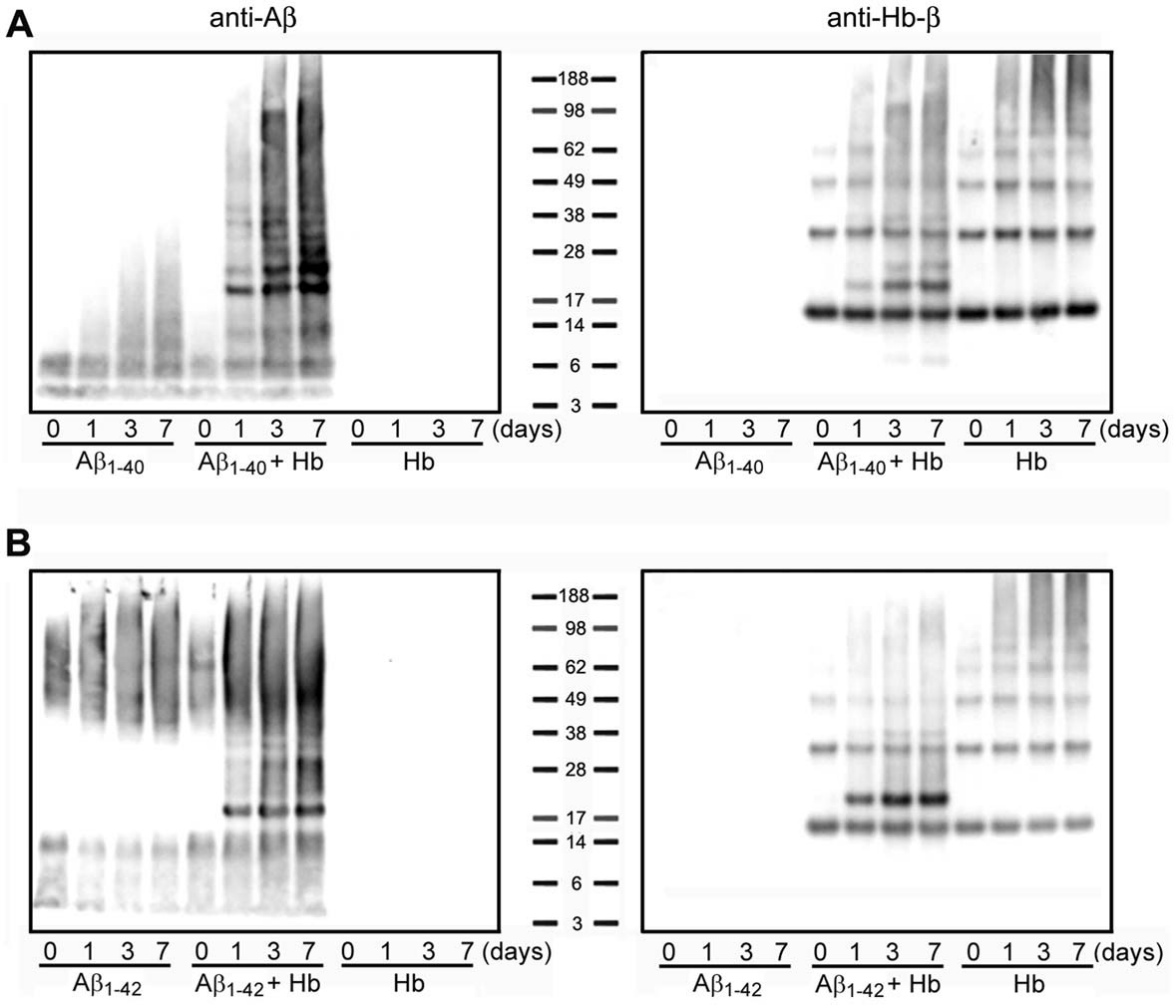
Interaction between hemoglobin (Hb) and Aβ. Freshly dissolved Aβ peptides (50 µM) were incubated with an equal mass of Hb (3.5 µM) at 37°C for 0, 1, 3 and 7 d. **A**) Aβ_1–40_; **B**) Aβ_1–42_. Left panels, anti-Aβ; right panels, anti-Hb-β chain.

To demonstrate the interaction between Hb and Aβ, equal masses of Hb and Aβ were incubated at 37°C for 0, 1, 3 and 7 d. Western blotting revealed that, under reducing condition, freshly dissolved Aβ_1–40_ primarily migrated as 4 kDa monomers, 8 kDa dimers and 12–16 kDa trimers/tetramers (Fig. 1A, left panel); whereas, freshly prepared Aβ_1–42_ was readily aggregated at the start of incubation and formed smear signals between 4∼12 kDa and large oligomers around 48–96 kDa (Fig. 1B, left panel). The amount of Aβ_1–40_ oligomers (Fig. 1A, left panels) and Aβ_1–42_ oligomers (Fig. 1B, left panels) increased as incubation time increased. Hb, a 64 kDa tetramer with two 16 kDa each of Hb-α and Hb-β subunits, was dissociated into 16 kDa monomers, 32 kDa dimers and 48 kDa trimers (Fig. 1, right panels). When Hb co-incubated with Aβ, large Aβ^+^ (Fig. 1, left panels) and Hb^+^ (Fig. 1, right panels) aggregates were evident. Among the large aggregates, discrete bands corresponded to Hb monomer and Aβ monomer (20 kDa), Hb monomer and Aβ dimer (24 kDa), Hb dimer and Aβ monomer (36 kDa) and Hb dimer and Aβ dimer (40 kDa) were noticed (Fig. 1). These results suggest that Hb binds to Aβ and enhances its aggregation.

### Heme is the critical Aβ binding moiety of Hb

**Figure 2:**
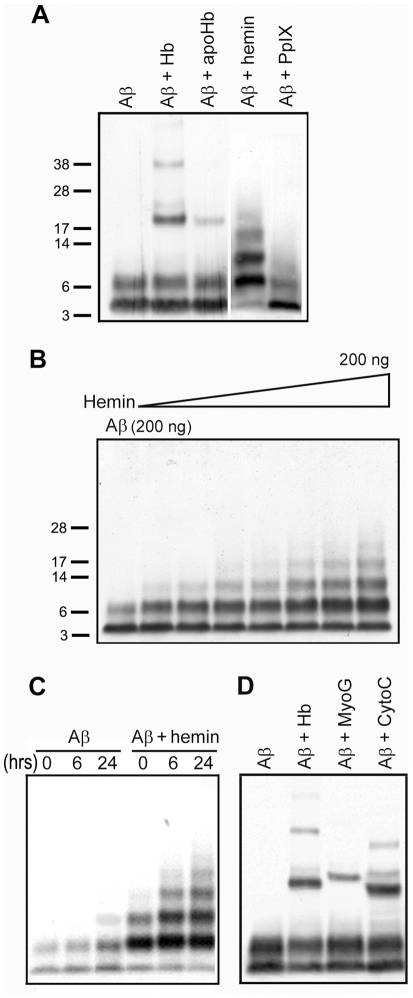
Heme is the Aβ binding moiety of hemoglobin (Hb). **A**) Interactions between Aβ and various part of Hb. **B**) Hemin induced the Aβ oligomerization in a dose-dependent fashion. **C**) Hemin induced the Aβ oligomerization in a time-dependent fashion. Incubation times: 0, 6, 24 hours. **D**) Aβ binds to Hb, myoglobin (MyoG) and cytochrome C (CytoC).

To characterize the Aβ binding domains of Hb, we separated the Hb into apoHb and heme. Our results showed that the discrete Aβ^+^ bands (Hb/Aβ complexes at 20, 24, 36 kDa, etc.) induced by Hb were greatly reduced when Hb was replaced by apoHb (Fig. 2A), suggesting the critical role of heme in the Hb-Aβ binding. As heme is a prosthetic group that consists of an iron contained in the center porphyrin, we then examined the effects of iron-free protoporphyrin IX (PpIX, porphyrin ring form of Hb-heme) and iron-containing hemin (the ferric salt of heme) on Aβ aggregation. As shown in Figure 2A, hemin effectively facilitated the formation of small Aβ oligomers, while iron-free PpIX lost the ability to interact with Aβ. The interaction of hemin and Aβ was further evaluated in dose- and time- dependent fashions. When a constant level of Aβ was incubated with various concentrations of hemin, the levels of SDS-stable Aβ dimers, trimers and tetramers increased as the amount of hemin increased (Fig. 2B). Similar results were obtained when the level of hemin was kept constant and the levels of Aβ were increased (data not shown). The formation of hemin-promoted Aβ aggregations was also increased as incubation time prolonged (Fig. 2C).

We were also interested in the Aβ-binding activity of other heme-containing hemoproteins. We chose myoglobin (MyoG) and cytochrome C (CytoC) because MyoG, like Hb, contains heme B, while CytoC contains heme C. Heme C differs from heme B in that the two vinyl side chains are covalently bound to the apoprotein through thioether linkages, whereas heme B is attached to the surrounding apoprotein through a single coordination bond between the heme iron and an amino acid side chain [25]. Our results showed that, in addition to Hb, both MyoG and CytoC interacted and formed larger complexes with Aβ (Fig. 2D).

**Figure 3:**
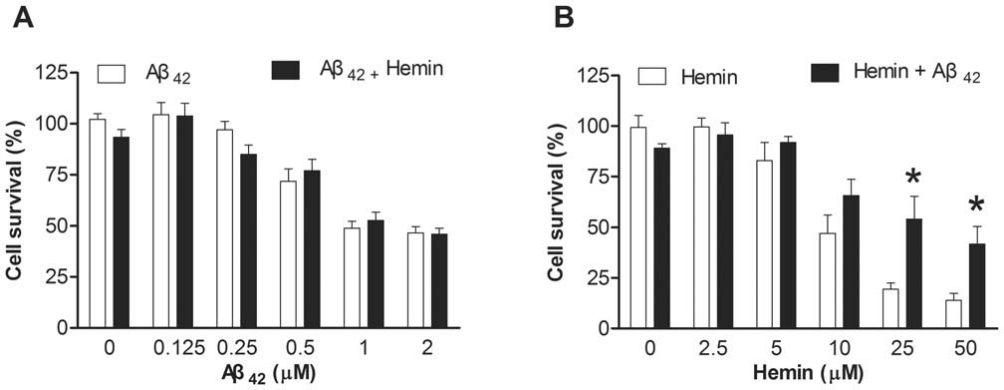
Effects of Aβ on hemin induced cytotoxicity. **A**) Differentiated neuroblastoma cells, SH-SY5Y, were treated with different doses of pre-incubated Aβ_1–42_ without (open bars) or with 0.25 µM of hemin (closed bars). **B**) Differentiated SH-SY5Y cells were treated with different doses of hemin without (open bars) or with 0.25 µM of pre-incubated Aβ_1–42_ (closed bars). The cell viability was determined by MTT reduction assay. Bonferroni's post-hoc test: * *p*<0.05 vs. respective hemin group.

We then examined the effects of hemin on the neurotoxicity of Aβ or vice versa on human neuroblastoma SH-SY5Y cells because both molecules are known to cause neuronal death. In our experimental settings, the LD_50_ of Aβ_1–42_ was approximately 1 µM (Fig. 3A), while the LD_50_ of hemin was about 10 µM (Fig. 3B). The presence of 2.5 µM of hemin, a concentration with no overt cytotoxicity, did not alter the LD_50_ of Aβ_1–42_ (Fig. 3A). On the contrary, 0.25 µM of Aβ_1–42_ reduced the cytotoxicity of hemin at high levels (Fig. 3B). Two-way ANOVA indicated that there were significant effects of hemin doses (*F* = 40.3, d.f. 5/48, *p*<0.001) and Aβ (*F* = 10.4, d.f. 1/48, *p* = 0.002) on the hemin-induced cytotoxicity. A significant interaction was evident suggesting that the hemin-induced cytotoxicity was influenced by Aβ. The protective effect disappeared when Aβ_1–42_ was replaced by an equal amount of a peptide composed of the reverse Aβ sequence (Aβ_40-1_, *F* = 1.2, d.f. 1/36, *p* = 0.272).

### Aging and APP/PS1 transgenes increase the levels of Hb in brain

**Figure 4:**
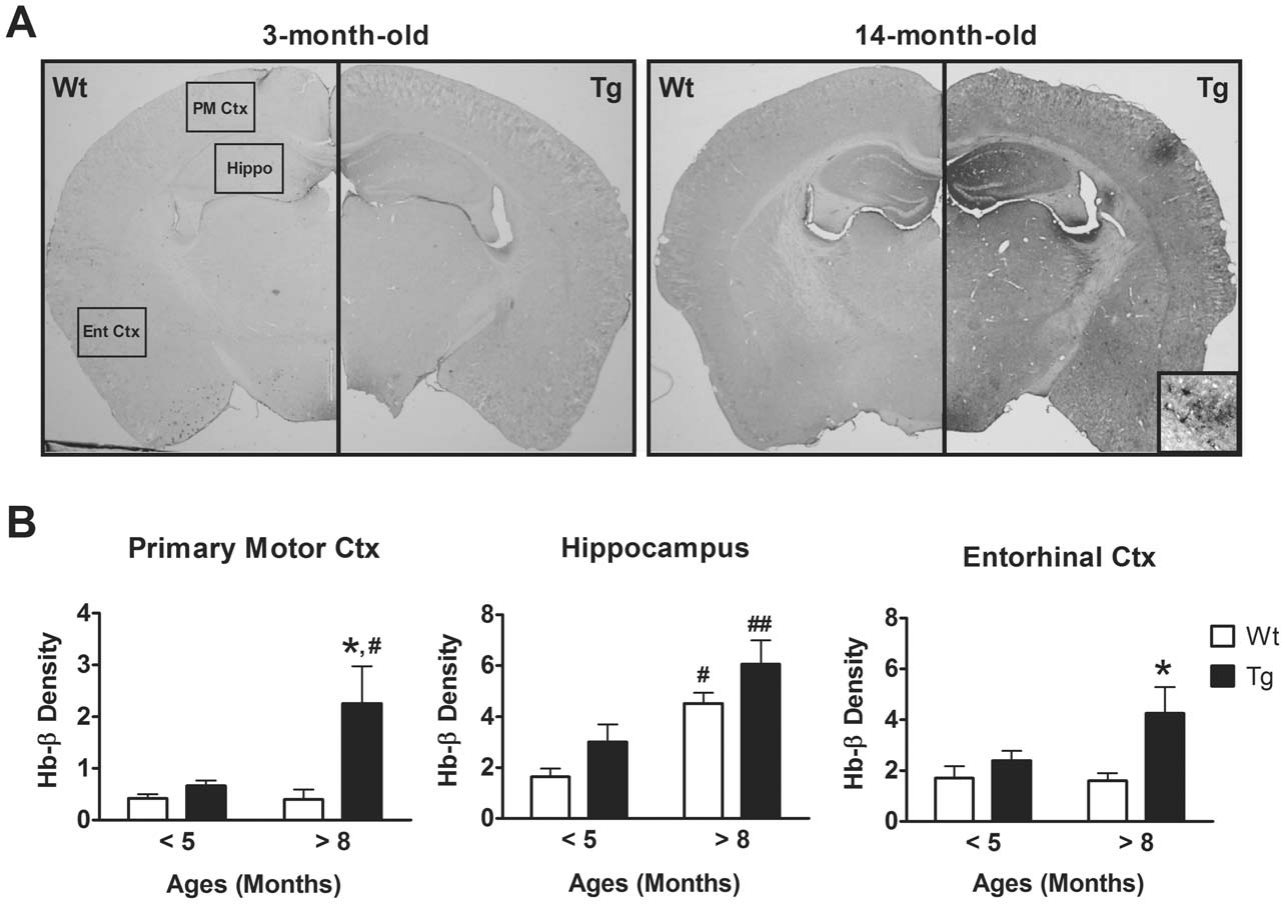
The expression of hemoglobin (Hb) in APP/PS1 transgenic (Tg) mice and wild-type (Wt) littermates. **A**) Representative immuno-micrographs reveal the intensities of Hb-β^+^ stains in the coronal brain sections of a 3-month-old and a 14-month-old Tg and Wt mice, respectively. Insert: enlarged micrograph shows the presence of Hb-β^+^ stains in a plaque-like structure and some brain cells. B) Quantitative results of the optical densities of Hb-β^+^ stains in primary motor, hippocampus and entorhinal cortex of <5-month-old and >8-month-old Tg and Wt mice. Bonferroni's post-hoc test: * *p*<0.05 vs. respective Wt group; # *p*<0.05, ## *p*<0.01 vs. respective <5-month-old group.

The effects of age on the expression of Hb in the brains were determined in APP/PS1 tg mice and their wild type (wt) littermates. Because the deposition of amyloid plaques in the APP/PS1 tg mice occurs around 6 months of age, we divided the mice into two age groups: <5 and >8 months. As shown in Figure 4A, the intensities of Hb-β^+^ signal were highest in the old APP/PS1 tg mice. We quantified the intensities of Hb-β^+^ signal in primary motor cortex (part of frontal cortex), hippocampus and entorhinal cortex (part of temporal cortex) because of their intimate associations with AD pathologies [26]. Two-way ANOVA indicated that there were significant effects of the APP/PS1 transgenes on the intensities of Hb-β^+^ signal in the primary motor (Fig. 4B, *F* = 5.0, d.f. 1/25, *p* = 0.035) and entorhinal cortices (Fig. 4B, *F* = 5.4, d.f. 1/25, *p* = 0.028); a marginal effect in the hippocampus was also evident (Fig. 4B, *F* = 3.7, d.f. 1/25, *p* = 0.065). Furthermore, the age of the mice also increased the intensities of Hb-β^+^ signal in the hippocampus (Fig. 4B, middle panel; *F* = 15.6, d.f. 1/25, *p*<0.001), although such effect was not presented in the primary motor (*p* = 0.108) and entorhinal cortices (*p* = 0.233).

**Figure 5:**
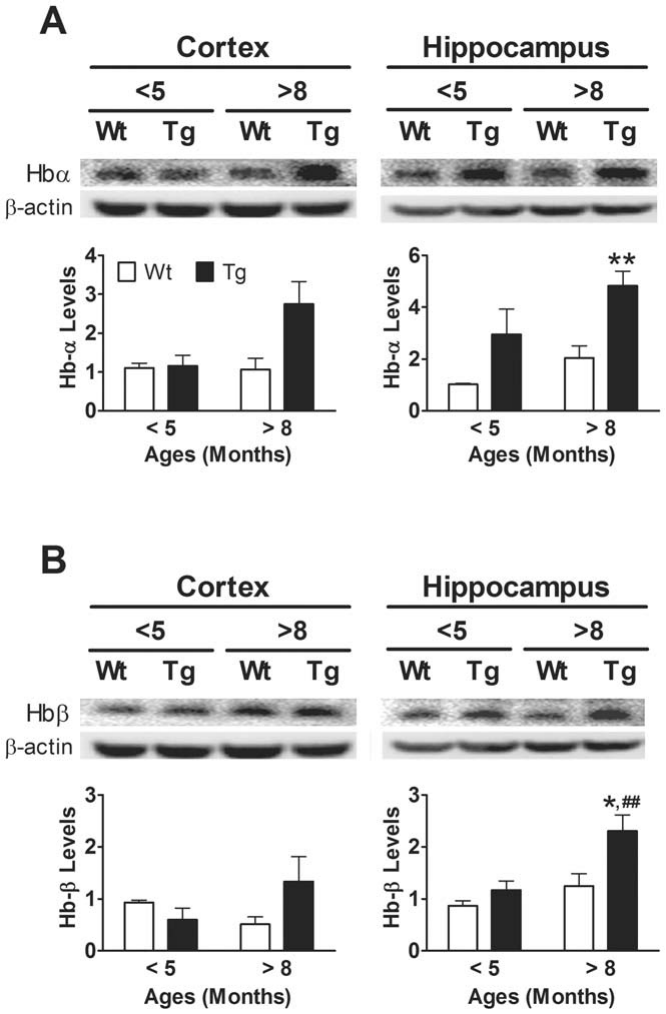
The levels of hemoglobin (Hb)-α and Hb-β in <5-month-old and >8-month-old APP/PS1 transgenic (Tg) mice and wild-type (Wt) littermates. Representative immunoblots are shown on the top of each panel whereas quantitative results are shown in the respective lower panel. The levels of Hb monomer (∼16 kDa) are quantified by immunoblotting analyses. Bonferroni's post-hoc test: * *p*<0.05, ** *p*<0.01, vs. respective Wt group; ## *p*<0.01 vs. respective <5-month-old group.

The levels of Hb were also quantified using immunoblotting analyses. As in the in vitro study shown in Figure 1, brain-derived Hb was also resolved as monomer, dimer, trimer and tetramer under denaturing conditions. This finding suggests that at least some of the brain Hb proteins are in their mature form. As the monomer band was predominant, we only quantified the relative band density of this form of Hb (Fig. 5, upper panels). The levels of Hb-α were up-regulated in the hippocampus, but not in the cortex, of the APP/PS1 tg mice relative to the wt mice (Fig. 5A, Two-way ANOVA, Cortex: *F* = 2.0, d.f. 1/25, *p* = 0.175; Hippocampus: *F* = 11.6, d.f. 1/25, *p* = 0.002). A similar effects of APP/PS1 tg on Hb-β were also evident (Fig. 5B, Cortex: *F* = 0.2, d.f. 1/25, *p*>0.5; Hippocampus: *F* = 4.3, d.f. 1/25, *p* = 0.049). Furthermore, the levels of Hb in the hippocampus, but not cortex, were also affected by age (Fig. 5; Two-way ANOVA, Hb-α: *F* = 4.4, d.f. 1/25, *p* = 0.047; Hb-β: *F* = 5.2, d.f. 1/25, *p* = 0.032). There was no significant interaction between age and the two brain regions. These results indicated that Hb was up-regulated by age and the overexpression of APP/PS1 genes.

### Localization of Hb in brains

**Figure 6:**
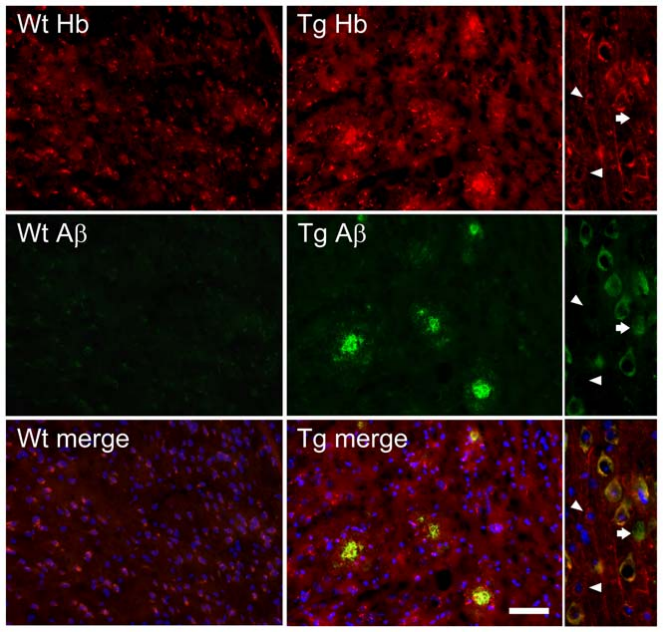
Localization of hemoglobin (Hb) immunoreactivity in brains of APP/PS1 transgenic (Tg) mice. Double immunofluorescent micrographs reveal the presence of Hb-β^+^ stains in Aβ^+^ amyloid plaques and some brain cells. Scale bars: 50 µm. Examples of enlarged micrographs are shown in the right. Arrowheads point to Hb-β^+^, but APP/Aβ^−^ cells; while, arrows show APP/Aβ^+^, but Hb-β^−^ cells.

While scrutinizing the brains of APP/PS1 tg mice, we found that Hb-β antibodies stained numerous plaque-like structures and cells (Fig. 4A, insert). These observations led us to investigate the distribution and localization of Hb in brains. We examined the expression of Hb in the brains of APP/PS1 tg mice because, under the control of prion promoter, neurons of the APP/PS1 tg mice generate large amount of Aβ intracellularly and extracellularly [27]. Double immunofluorescence revealed that, in the cortex and hippocampus of the APP/PS1 tg mice, the Hb-β^+^ plaque-like structures co-localized with Aβ^+^ amyloid plaques (Fig. 6). Furthermore, Hb-β was expressed not only in the APP^+^/Aβ^+^ neurons, but also in the cells without Aβ accumulation (Fig. 6, arrowheads in right enlarged panels). Conversely, we have also noticed that some neurons expressed abundant APP/Aβ in the cytoplasm, yet no or little Hb signal was detected (Fig. 6, arrows in right enlarged panels).

**Figure 7:**
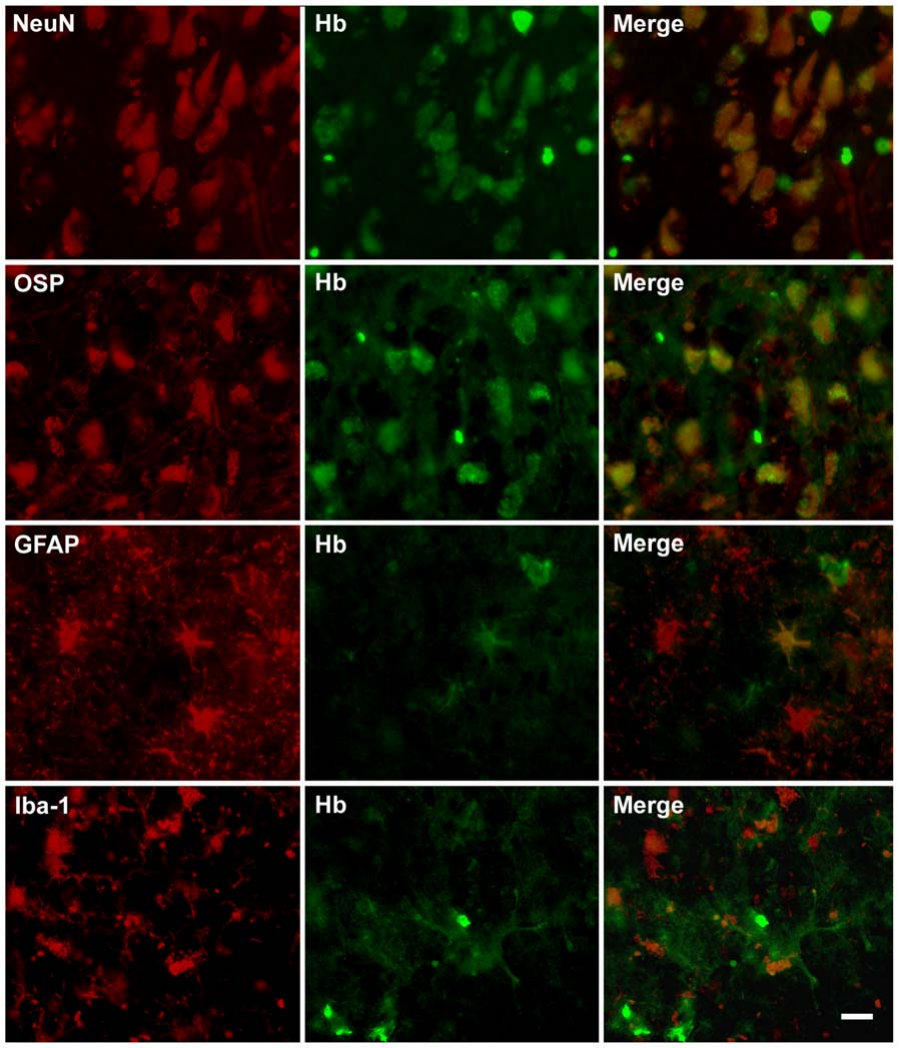
The distribution of hemoglobin (Hb) immunoreactivity in inferior temporal gyrus of post-mortem human brain. Hb-β^+^ stains are present in most NeuN^+^ neurons and OSP^+^ oligodendrocytes; while only a small fractions of GFAP^+^ astrocytes and Iba-1^+^ microglia express Hb. Scale bars: 10 µm.

To verify the identities of the Hb-expressing cells, we immunstained human postmortem brain slices with Hb antibodies and neuron (NeuN) or glia markers (oligodendrocyte: OSP, astrocyte: GFAP, microglia: Iba-1). Hb immunoreactivities were evident in a subpopulation of hippocampal and cortical neurons (Fig. 7). Furthermore, almost all the OSP^+^ oligodendrocytes were Hb-β^+^ (Fig. 7). In contrast, very few GFAP^+^ astrocytes and Iba-1^+^ microglia stained positive with Hb antibody (Fig. 7).

### Aβ surrounds the Hb in the brain

**Figure 8:**
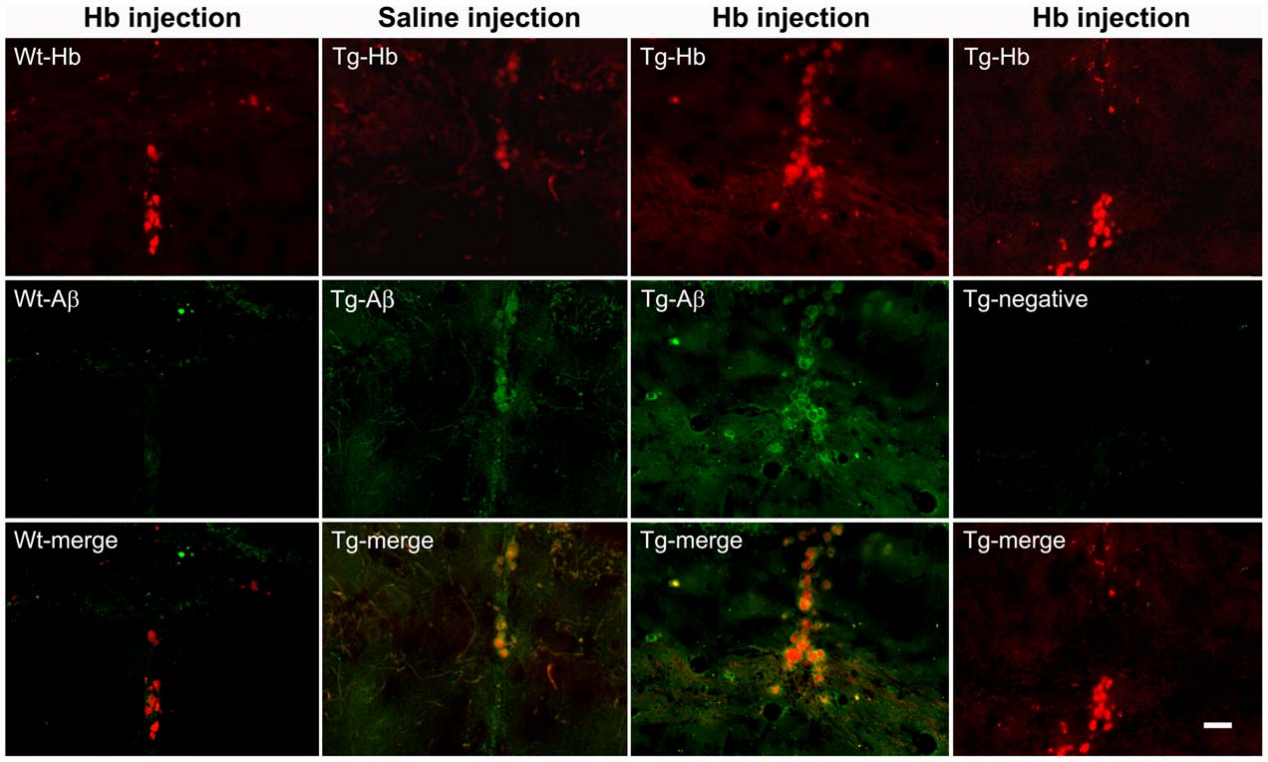
Hemoglobin (Hb) induces Aβ accumulation in the immediate surroundings of Hb. Human Hb (1 µl, 50 µg/µl) was injected into the dorsal hippocampus of 4-month-old APP/PS1 transgenic (Tg) mice. One week after the injection of Hb or saline, the brain sections were double stained for Hb-β and Aβ. Tg-negative: The sections were stained only by Hb antibodies, but not Aβ antibodies and serve as negative controls for Aβ staining. Scale bars: 30 µm.

To investigate the interaction between Hb and Aβ in vivo, we microinjected human Hb (1 µl, 50 µg/µl) into dorsal hippocampus of 4-month-old APP/PS1 tg mice. One week after the injection, we found that Aβ accumulated in the Hb injection site with Hb droplets in the center and Aβ surrounded in the periphery (Fig. 8). A similar effect was also evident in the saline-injected hippocampus of the APP/PS1 tg mice (Fig. 8), as microinjection inevitably induced a variable degree of hemorrhage along the needle track. We were unable to distinguish the patterns of Aβ accumulation around the injected human Hb from the injection-induced leakage of mouse Hb. This effect was not observed in wt littermates (Fig. 8).

## Discussion

In previous studies, we and others have identified human Hb as a potent Aβ-binding protein in the brain [23]. Further, we showed that Hb co-localized with Aβ in amyloid plaques and the cerebral amyloid angiopathy of post-mortem AD brains. In this study, we found that the interaction between Hb and Aβ was heme dependent. Other heme-containing proteins, such as myoglobin and cytochrome C, also interacted with Aβ. The iron atom within the protoporphyrin IX was indispensable for the binding of heme to Aβ. Heme-induced cytotoxicity was reduced by Aβ, presumably by the Aβ-iron interaction. Hb was present in amyloid plaques and brain cells, mainly in neurons and oligodendrocytes. Furthermore, the levels of Hb increased in aging and APP/PS1 tg mice. Finally, we showed that injection of exogenous human Hb into the brains of APP/PS1 tg mice induced Aβ accumulation at the site of Hb deposition.

Hemoglobin is expressed in specific brain cells and the expression levels of brain Hb increased with age in APP/PS1 tg mice. The presence of Hb-α and Hb-β mRNAs in brains was initially reported by Ono and Cutler more than three decades ago [28]. They found that both the levels of Hb-α and Hb-β mRNA were elevated in the cytoplasm of aged brain cells in rats [28]. The presence of Hb mRNA in brain tissue has been confirmed in rodents and humans [23], [29]–[32]. Interestingly, the levels of Hb were not only positively correlated with age in rats, but negatively correlated to their learning and memory performances [29]. Hemoglobin proteins and Hb-derived peptides have been isolated from human CSF and brain tissues using different biochemical techniques [24], [33]–[36]. The expression of Hb mRNA was evident in the neurons of rat cortex, hippocampus and substantia nigra by in situ hybridization [32]. These findings have been confirmed by immunohistochemical staining of Hb in the neurons of human and rodent brains [23], [31], [32], [34]. The expressions of Hb in oligodendrocytes and astrocytes have also been documented previously, but with some controversy [31], [37], [38]. Taken together, these findings demonstrate that Hb is expressed in brain cells and that the levels of brain Hb increase with age.

The levels of Hb were also elevated in the brains of AD patients. In a previous publication, we have shown that both the Hb mRNA and protein levels were up-regulated in the AD brains, with the levels of Hb highest in the hippocampus and cortical gray matter, followed by white matter and the lowest in cerebellum [23]. We have also shown that 64% to 96% of amyloid plaques and almost all the cerebral amyloid angiopathy were positively stained for Hb [23]. In this study, we further confirm that Hb is present in the extracellular amyloid plaques and cytoplasm of brain cells. Using proteomic approaches, elevated Hb levels were found in temporal and sensorimotor cortices and CSF of AD patients [35], [36].

Exogenous Hb released from red blood cells after traumatic brain injury or intracerebral hemorrhage is highly neurotoxic [39], [40]. However, recent evidence indicates that enhanced expression of intracellular globins, such as neuroglobin [41]–[43] and haptoglobin [44], protects neurons against hypoxic-ischemic injury. Considering that: 1) Hb is the most abundant oxygen-carrier protein in the human body, 2) the gene expression of Hb is regulated by hypoxia-inducible factor 1α [45] and 3) cerebral hypoperfusion is frequently observed in aged brains [46], it is possible that an up-regulation of Hb during aging is a feedback response to hypoxia. Indeed, enhanced Hb expression in neurons has been demonstrated to be regulated by hypoxia-stimulated erythropoietinin in rodents [38]. Furthermore, Aβ is also known to increase vascoconstriction and decrease cerebral blood flow [47], [48]. Aging associated hypoperfusion and increased Aβ could stimulate the production of an intracellular pool of Hb that may serve as an oxygen reservoir during periods of increased oxygen demand [38].

A portion of the brain Hb may be derived from the transient extravasation of erythrocytes or free-Hb in the neuropil originating from the peripheral circulation. Accumulating evidence indicates that aging and AD are associated with profound changes in blood-brain barrier structure and function [46], [49], [50]. In addition, cerebrovascular diseases commonly observed in AD, including cerebral amyloid angiopathy, microvascular degeneration (capillary tortuosity, string vessels, vascular fibrohyalinosis and lipohyalinosis), microinfarcts, subcortial lacunes and micro-hemorrhages, are known to disturb the integrity of cerebral vessels and alter brain perfusion [51]–[54].

In this study, we also demonstrate that the iron-containing heme group is critical for Aβ binding to Hb. The binding of Aβ to heme has been previously characterized by Atamna's group [55], [56]. In addition, we found that other heme-containing proteins were likewise capable of interacting with Aβ, reinforcing the view that the iron-containing heme group mediates the interaction between Aβ and hemoproteins. Hb and other hemoproteins are potentially hazardous because their iron-containing heme can trigger a Fenton reaction capable of catalyzing the generation of the hydroxyl-radical [40], [57]. High levels of Aβ, especially the oligomeric forms, are also known to be neurotoxic in vitro [5]–[8]. However, the Aβ-induced neurotoxicity was not recaptured in most in vivo studies by directly injecting Aβ into the living brain [58]–[61]. Surprisingly, while iron has been shown to aggravate the neurotoxicity of high levels of Aβ, iron-induced oxidation, lipid peroxidation and neurotoxicity are blocked by Aβ [58], [62], [63]. The latter findings were supported by our observation that the cytotoxicity of heme was reduced by applying a sublethal dose of Aβ to the culture. These findings also suggest that by virtue of binding to Aβ, the hemorrhage-associated Hb/heme toxicity can be confined.

The interaction between Aβ and Hb in vivo suggests that Aβ may act as a protective adhesive and that its continuous accumulation may ultimately participate in the formation of amyloid plaques and/or cerebral amyloid angiopathy. However, as microinjection inevitably induced hemorrhage, we cannot exclude the possibility that other molecules in the blood also participate in the accumulation of Aβ along the injection-induced leakage. Formation of amyloid plaques at sites of capillary bleeding was supported by the findings that every plaque in AD brain is positive for Hb, heme, or vessel-derived proteins [64], [65]. Detailed analyses of these plaques have led to the conclusion that each amyloid plaque is either in direct contact or closely related to a small vessel [64]–[68]. Similarly, stereological analyses of APP tg mice also suggest that leaky capillaries create a ‘nidus’ for plaque formation [69]–[71]. The direct association of amyloid plaques with the microvasculature was characterized by vascular corrosion casts and scanning electron microscopic visualization of the entire brain vasculature in APP tg mice [72]. In young APP tg mice, before typical amyloid plaques appeared, microvascular alterations with small amyloid deposits attached to the distorted vessels are present [72]. In older APP tg mice and AD patients, some vessels are truncated with their stumps terminating close to amyloid plaques [64], [72], [73]. The putative leaking vessel-sealing function of Aβ could also explain why immediately after traumatic brain injury the production of Aβ is up-regulated [16], [17]. Therefore, it is not surprising that disruption of deposited Aβ from the vasculature by anti-Aβ antibodies significantly increased the frequency of intracerebral hemorrhage in APP tg mice [74]. As proposed previously, Aβ might form an intracranial “scab” maintaining the structural integrity of cerebral vasculatures [71], [75].

The idea that capillary hemorrhage is the key event in plaque formation has been proposed previously [76]. In his excellent review, Stone [76] provides rich pathological and epidemiological evidence supporting the association between microhemorrhage and amyloid deposition. Stone hypothesized that microhemorrhage-induced ischemia results in the production of Aβ and that the oligomerization of this molecule is elicited by blood components, such as Hb. The Aβ oligomers then assemble into fibers and amyloid plaques, that damage the neural tissues. Our observations that Hb binds Aβ and promotes the oligomerization favor his premise. However, our data also suggest that instead of inducing damage to neural tissues, sublethal Aβ levels seem to play a protective role in the early stages of hemorrhage. The associated secondary injuries, i.e. inflammation and oxidative stress, emerge at later chronic stages when the wound-healing scars gradually evolve into mature amyloid plaques. In light of these observations, we would like to propose the following scenario: 1) when microhemorrhage occurs as the result of aging, traumatic brain injury or cerebrovascular disease, Aβ can act as a sealant preventing further leakage of toxic blood components; 2) Aβ peptides aggregate around the leaked Hb limiting its toxicity; 3) Aβ binds to heme preventing the generation of toxic reactive oxygen species and promoting heme degradation [55]; 4) Aβ chelates and sequesters free iron thereby greatly reducing direct iron-elicited oxidative toxicity. Malfunction of the blood-brain barrier is prevalent in aged individuals and the observed physical proximity of plaques and capillaries suggests that some amyloid deposits may result from the chronic accretion of Aβ around sites of vascular leakage or damage.

## Materials and Methods

### Materials

Recombinant Aβ_1–40_ and Aβ_1–42_ peptides were purchased from rPeptide (Bogart, GA, USA). The composition and the purity of the peptides were characterized by HPLC and mass spectrometry. Human Hb, myoglobin, cytochrome C, hemin and PpIX were purchased from Sigma-Aldrich (St. Louis, MO, USA). The preparation and isolation of apoHb, the heme-free Hb, was carried out using the acid-acetone method as previously described [77]. HPLC-grade dimethyl sulfoxide (DMSO, 99.9%), Tris-HCl, SDS and chloral hydrate were purchased from Sigma-Aldrich.

### Binding Assay

Recombinant Aβ_1–40_ or Aβ_1–42_ powders (1 mg) were suspended in 1 ml of 1% NH_4_OH and diluted in 50 mM of Tris-HCl, pH 7.4, to make an Aβ working solution of 100 µM. The Aβ working solution was incubated with an equal volume of Hb solution (6.8 µM in DDH_2_O) and incubated at 37°C for 0, 1, 3 and 7 d. The final concentrations in the solution were 50 µM for Aβ and 3.5 µM for Hb (1∶1 in weight ratio). Hemin (MW 651.9) and PpIX (MW 562.7) were suspended in 100% DMSO (0.5 mg/ml) and diluted in 50 mM of Tris-HCl, pH 7.4, to make the desired amounts. After mixing with Aβ peptides, the mixtures were incubated at 37°C for 0, 6 or 24 h. At the end of the incubation, 12 µl of the solutions were mixed with 4 µl of 4× sample buffer (Invitrogen, Carlsbad, CA, USA), heated to 70°C for 10 min and resolved on a 4–12% Bis-Tris gel (Nu-PAGE, Invitrogen). The resolved proteins were transferred onto nitrocellulose membranes (GE Healthcare Bio-Sciences Corp., Piscataway, NJ, USA), briefly boiled in 50 mM Tris-HCl buffer, pH 7.4, blocked with 5% non-fat milk, and probed with a mixture of anti-Aβ monoclonal antibodies (4G8, 1∶10000; 6E10, 1∶10000; Covance, Princeton, NJ, USA). A parallel gel was used to probe with anti-Hb-β chain goat polyclonal antibodies (M-19, Santa Cruz Biotech.; 1∶500).

### Animals

All experiment protocols were carried out in accordance with the National Institutes of Health Guidelines for animal research (Guide for the Care and Use of Laboratory Animal) and approved by the National Cheng Kung University Institutional Animal Care and Use Committee. Male APP/PS1 double tg mice were purchased from the Jackson Laboratory (Bar Harbor, ME) and maintained in a controlled environment (temperature 23±1°C; 12 h light/12 h dark cycle, light cycle begins at 06:00) of the Laboratory Animal Center of National Cheng Kung University with unrestricted access to food and water. The APP/PS1 double tg mice express a chimeric mouse/human amyloid precursor protein (Mo/HuAPP695swe) and a mutant human presenilin 1 (PS1-dE9) with the exon 9 deletion under the control of mouse prion promoter elements. Shortly after weaning, these mice were genotyped with PCR using a protocol provided by the Jackson Laboratory.

### Surgery

Male APP/PS1 tg mice at 4-month-old (20 g–25 g) were anesthetized by 0.4 g/kg of chloral hydrate (Sigma) before the procedure. The mouse skull was mounted on a stereotactic apparatus (Stoelting Co., IL) and a midline incision of the scalp was done to expose the skull. The microinjection was performed at the dorsal hippocampus (stereotaxic coordinates (mm from bregma): anterior/posterior, -1.7; lateral, 1.2; dorsoventral, 1.3) using a 29-gauge needle attached to a 10-µl syringe. One µl of Hb (50 µg/µl) was injected and the injection rate was set at 0.2 µl/min. An equal amount of normal saline was infused to serve as control. The needle was removed 5 min after the completion of injection.

### Tissue preparations

After deep anesthesia, mice were intracardially perfused with paraformaldehyde and their brains were removed, post-fixed and sliced at 30 µm as previously described [78]. Coronal sections were collected in cryoprotectant and stored at −20°C. For biochemical analyses, freshly removed brains were dissected and rapidly submerged in liquid nitrogen for 10 min before storage at −70°C. The paraformaldehyde fixed, free floating frozen sections (40 µm in thickness) prepared from the right temporal lobe and associated cortex from both AD and ND individuals were kindly provided by Dr. Tom Beach (Brain Bank of the Banner Sun Health Research Institute, Sun City, AZ, USA).

### Immunohistochemistry

The paraformaldehyde-fixed brain sections were preblocked in 5% normal rabbit serum (Vector Laboratories, Burlingame, CA) and stained with anti-hAPP mouse monoclonal antibodies (2C12, Sigma; 1∶500), anti-Aβ mouse monoclonal antibodies (4G8; 1∶500) or anti-Hb-β goat polyclonal antibodies (M-19; 1∶500). The specificity of the Hb antibody has been demonstrated previously by pre-absorption with Hb in human brain sections [23]. The brain sections were then incubated with appropriate peroxidase-conjugated secondary antibodies and an avidin-biotin peroxidase (Vectastain Elite ABC kit, Vector) using 3,3′-diaminobenzidine as the substrate.

### Immunofluorescence

For immunofluorescence double labeling, free-floating tissues were washed three times in PBS with 0.5% Triton X-100 and stained for Hb-β (M-19; 1∶250), Aβ (4G8; 1∶250), NeuN (Millipore, Billerica, MA; 1∶500), OSP (Abcam, Acmbridge, MA; 1∶500), GFAP (Dako, Glostrup, Denmark; 1∶500) and Iba-1 (Wako, Osaka, Japan; 1∶500) antibodies overnight at 4°C. For detection of primary antibodies, fluorescein Alexa488-conjugated anti-goat (Invitrogen; 1∶1000), FITC-conjugated (Jackson ImmunoResearch, West Grove, PA; 1∶1000) anti-mouse, Rhodamine-conjugated anti-rabbit (Jackson ImmunoResearch; 1∶1000), or biotin-conjugated anti-goat (Jackson ImmunoResearch; 1∶1000) secondary antibody was used according to the instructions from the manufacturer. Control sections were treated identically except that the primary antibody was omitted. Coverslips were mounted in 2.6% DABCO (Sigma) in 90% glycerol/10% PBS and imaged with a Zeiss fluorescent microscope.

### Image analysis

To analyze the relative optical intensity of immunomicrographs, the photomicrographs were taken by an Axiocam MRc digital camera connected to a computer equipped with Axiovision 4.8 software (Carl Zeiss, Inc., Thornwood, NY). Signal relative intensities were obtained by dividing the optical densities of the desired brain regions to that of backgrounds of each section using the ImagePro plus 6.0 software (Bethesda, MD). The optical density of an area in the corpus callosum free of any signal was designed as background reading.

### Neurotoxicity assay

The human neuroblastoma SH-SY5Y cells were cultured in DMEM/F12 medium (Invitrogen) supplemented with 10% fetal bovine serum, 1% L-glutamine and 1% penicillin/Streptomycin. Cells were grown in a humidified atmosphere of 5% CO_2_ and 95% air at 37°C. Three days before treatment, 1×10^4^ cells were seeded in 96-well plates and differentiation was induced by 10^−5^ M retinoic acid in 200 µl serum-free DMEM media. The survival rates of the cells were determined by the MTT, 3-(4, 5-dimethylthiazol-2-yl)-2, 5-diphenyltetraolium bromide (Sigma), reduction assay. Aβ_1–42_, dissolved in 1% NH_4_OH and diluted in Tris-HCl as described above (Binding Assay section), was incubated at 37°C for 24 h before treating the cells. Hemin was prepared as aforementioned. After treating the differentiated SH-SY5Y cells with different concentrations of Aβ_1–42_, hemin or a mixture of Aβ_1–42_ and hemin for 48 h, 50 µl of MTT solution was added to each well for 4 h, followed by adding another 50 µl of DMSO per well with dark incubation for 20 min. Absorbency was measured by a spectrophotometer at wavelength of 595 nm.

### Statistics

Data were expressed as mean ± S.E.M. Two-way analysis of variance followed by Bonferroni posttest was used to analyze the results of Hb expression levels in brains with the APP/PS1 transgenes as within-subject factor and age as between-subject factor. The animal number used in each group was indicated by n.
